# Establishment and Investigation of a Multiple Gene Expression Signature to Predict Long-Term Survival in Pancreatic Cancer

**DOI:** 10.1155/2020/1570862

**Published:** 2020-09-15

**Authors:** Zhiqiang Zhang, Jiangning Gu, Menghong Yin, Di Wang, Chi Ma, Jian Du, Zhikun Lin, Siling Hu, Xuelong Wang, Ying Li, Guang Tan, Haifeng Luo, Gang Wei

**Affiliations:** ^1^CAS Key Laboratory of Computational Biology, CAS-MPG Partner Institute for Computational Biology, Shanghai Institute of Nutrition and Health, Shanghai Institutes for Biological Sciences, University of Chinese Academy of Sciences, Chinese Academy of Sciences, Shanghai, China; ^2^Department of Hepatobiliary Surgery, The First Affiliated Hospital of Dalian Medical University, Dalian, Liaoning Province, China; ^3^Department of Sports Medicine, Dalian Municipal Central Hospital, Dalian Medical University, Dalian, Liaoning Province, China; ^4^Department of Scientific Research, Eyes & ENT Hospital of Fudan University, Shanghai, China

## Abstract

Pancreatic cancer remains a lethal type of cancer with poor prognosis. Molecular classification enables in-depth, precise prognostic assessment. This study is aimed at identifying a robust and simple mRNA signature to predict the overall survival (OS) of pancreatic cancer (PC) patients. Differentially expressed genes (DEGs) between 45 paired pancreatic tumor samples and adjacent healthy tissues were selected. For risk determination, a LASSO Cox regression model with DEGs was used to generate the OS-associated risk score formula for the training cohort containing 177 PC patients. Another five independent datasets were used as the testing cohort to determine the predictive efficiency for further validation. In total, 441 DEGs were selected after considering the enrichment of classical pathways, such as EMT, cell cycle, cell adhesion, and PI3K-AKT. A five-gene signature for risk discrimination was established with high efficacy using LASSO Cox regression in the training group. External validation showed that patients identified by the gene expression signature to be in the high-risk group had poorer prognosis compared with the low-risk patients. Further investigation identified the differential epigenetic modification patterns of the five genes, which indicated their roles in tumor progression and their effect on therapy. In conclusion, we constructed a robust five-gene expression signature that could predict the OS of PC patients, offering a new insight for risk discrimination in daily clinical practice.

## 1. Introduction

Although great improvements have been achieved in detection and treatment of many types of highly malignant tumors, such as lung and breast cancers, the overall survival (OS) and prognosis of pancreatic cancer (PC) remain poor, with a five-year survival rate of only around 8% [1]. Surgical resection offers the only chance for long-term survival, since PC is naturally resistant to chemo- and radiotherapy. Only about 20% of patients have the opportunity to receive surgical resection, and the median OS is only around 24 months [2]. In daily clinical practice, TNM staging formulated by AJCC is used to determine the course of the treatment. However, even two patients at the same TNM stage may have totally different prognoses [3]. This means that clinical histopathological classification has inherent limitations in predicting the prognosis for PC patients, and thus, identification of new biomarkers for prognostic assessment is urgently required [4].

With the development of next-generation sequencing, genetic markers have been pursued for cancer classification, and these have come to play an important role in the assessment of prognosis and the best course of treatment. The term “molecular subtypes” refers to tumors with similar morphology but with very different clinical features. Although molecular subtyping is a highly complex system, it is the major diagnostic and prognostic strategy in clinical practice [5]. For instance, breast cancer is divided into different subtypes based on the markers ER, PR, and HER2, and each subtype is associated with different treatment modalities and overall survival. A similar pattern has been determined for EGFR and ALK in non-small-cell lung adenocarcinoma [6, 7]. However, the much-needed investigation of PC subgroups is still in its infancy. Scholars previously attempted to classify PC according to the expression patterns of single genes based on studies which showed the relevance of these genes to OS. However, despite the promising progress in the laboratory, no significant improvement has been achieved in the clinic.

Transcriptomic sequencing has provided new opportunities and already helped make some achievements, in PC classification. On the one hand, Collisson et al. classified PC into three subtypes based on a 62-mRNA gene expression signature and named them classical, quasimesenchymal, and exocrine-like tumors. These three groups differed in the survival time of the patients and sensitivity to chemotherapeutics [8]. On the other hand, Moffitt et al. divided PC into two subtypes, basal-like and stromal, of which the former one had a worse prognostic outcome. Stromal subtypes were further divided into two groups, “normal” and “activated,” which immensely differed from each other in terms of their prognosis [9]. Based on these two studies, Bailey et al. used RNA-seq of 96 genes and 232 microarray data and identified ten key signaling pathways in PC. They then accordingly divided PC into four subtypes—squamous, immunogenic, pancreatic progenitor, and ADEX [10]. Wartenberg et al. classified PC into three subtypes according to their immune status: immune-rich, immune-exhausted, and immune-escape. These three groups greatly differed from each other in their prognostic outcomes [11].

Molecular subtype classification based on gene expression signatures can be used for prognostic and therapeutic assessment, including surgery and chemotherapy options, even potentially during early stages of the disease. In this respect, there has already been some progress in other tumor types. For instance, Li et al. established a four-miRNA signature for predicting trastuzumab's effect on HER2-positive breast cancer patients [12]. Zhou et al. established a seven-miRNA detection system for early diagnosis of hepatocarcinoma, and this system is currently in use for diagnostic assessment in clinics [13]. However, a similar approach has not been comprehensively pursued for PC, and there have been only a few studies. Taking this into consideration, we used meta-analysis on pooled datasets, totaling 875 PC patients' samples. The entire dataset included transcriptomic sequencing data, survival information, and epigenetic background. Using this, we determined a multigene expression signature that predicts the OS and analyzed the mechanism underlying this pattern for the development of potential therapies in the future.

## 2. Materials and Methods

### 2.1. Data Availability

The raw gene expression data and corresponding clinical information of pancreatic patients were downloaded from the Gene Expression Omnibus (GEO, https://www.ncbi.nlm.nih.gov/gds/). The processed TCGA data was derived from UCSC-Xena (https://xenabrowser.net/). These samples had been profiled using whole-genome DNA microarray (Affymetrix or Agilent) and RNA-seq (Illumina). The datasets contained 875 patient data, including 555 patients with available survival data. The dataset of TCGA was used as the training cohort after removing the patients that lacked survival data information. The dataset of GSE28735 was processed to obtain the differentially expressed genes between pancreatic cancer and adjacent normal tissues. The datasets of GSE21501, GSE57495, GSE62165, GSE62452, GSE79668, and Bailey et al., 2016 served as the independent validation cohorts. The information about all the datasets is shown in Table 1 and Supplementary Table 3.

### 2.2. Normalization and Annotation of GEO Data and TCGA Data

First, we normalized each DNA microarray-based dataset using the Robust Multichip Average (RMA) method for the raw Affymetrix data derived from GEO. Then, we mapped hybridization probes across the different technological platforms with the corresponding SOFT-formatted family files in R. When multiple probes were mapped to the same gene symbol, we calculated the average expression of the genes in the dataset. For the data from the Agilent; TCGA; Bailey et al., 2016; GSE62165; and GSE79668 datasets, we used the available normalized data. The datasets above were log2-transformed.

### 2.3. Selection of the Differentially Expressed Genes and Construction of LASSO Cox Regression Model

The GSE28735 dataset consisted of 45 pairs of pancreatic tumor and adjacent healthy tissues. We used these datasets to identify the differentially expressed genes. The genes with significant expression differences were defined based on the following parameters: FDR < 0.05, ∣log2fc | >1. Having identified 441 differentially expressed transcripts, we sought to establish an association with patient outcomes. Then, we merged the differently expressed gene list derived from the GSE28735 dataset with the TCGA (*n* = 177) dataset to generate the training cohort. Next, the logical regression analysis with Least Absolute Shrinkage and Selection Operator (LASSO) was applied to select the gene expression signature [14], which is a selection method that handles the high-dimensional regression variables with no prior feature selection step by shrinking all regression coefficients toward zero and thus forcing many regression variables to be exactly zero. The penalty regularization parameter lambda was chosen via 10-fold cross-validation cv. glmnet, which is implemented in the R package glmnet [15]. The lambda was finalized using the lambda.min = 0.2215, which is the value of lambda giving the minimum mean cross-validated error. Finally, we obtained five-gene expression signatures and corresponding coefficients.

### 2.4. Establishment of the Risk Score Formula

Based on the expression levels of the five genes, a formula was constructed to calculate an OS risk score for each patient as follows:

(1) $$\begin{array}{c} \text{Risk score}=\overset{N}{\underset{i=1}{\sum }}\text{E}\text{xp}\left(i\right)*\text{Coefficient}\left(i\right). \end{array}$$

In our risk score formula, *N* (*N* = 5) is the number of genes, Exp is the expression value of each gene, and Coefficient is their corresponding coefficient from the LASSO Cox regression. In this case, we would be able to generate a risk score for each patient, from which patients could be divided into high- and low-risk score groups with an optimal cutoff score determined by X-tile plots [16] based on the association with OS.

### 2.5. Receiver Operating Characteristic (ROC) Curve Analysis

Based on the LASSO Cox regression, a group of four genes were selected; ROC was employed to demonstrate the sensitivity and specificity of different variables by risk score. The prognosis performance was evaluated using a time-dependent receiver operating characteristic (ROC) curve analysis [17]. In order to evaluate the predictive accuracy and robustness of our prognostic model, AUC at 1 year, 2 years, and 3 years was calculated in the training and different validation cohorts according to the five-gene expression signature. The spanning parameter of the NNE approach was span =0.25 × nobs^−0.20^, which was performed in R package survivalROC [17].

### 2.6. Overall and Stratified Survival Analysis

According to the risk score formula, we divided each patient into high- or low-risk groups with the optimal cutoff value derived from the training cohort. The Kaplan-Meier method was used to assess the difference in the survival rates of high- and low-risk patients. Then, univariate and multivariate Cox regression survival analysis was performed to evaluate the various clinicopathological features, such as age, gender, tumor stage, and grades. Moreover, to further explore the impact of clinical pathological features on the value of risk score, stratified survival analysis related to age at the time of diagnosis (>60 or ≤60), gender (male or female), AJCC stage (I/IIA or IIB/III/IV), T stage (T1/T2 or T3/T4), N stage (N+ or N-), and histological grade (G1/G2 or G3/G4) was conducted. A *P* value < 0.05 according to the log-rank test was considered significant. The hazard ratio (HR) and 95% confidence interval (CI) were calculated. All of these statistical analyses were performed in R or corresponding R packages survival and survminer.

### 2.7. Pathway Enrichment Analysis

The enrichment analysis was performed to predict the biological processes and KEGG pathways of the DEGs in an online tool—Metascape [18]. The GSEA was shown to predict the hallmarks of the tumor and healthy pancreas enrichment [19]. Both DEGs and GSEA input data were derived from GSE28735.

### 2.8. Epigenetic Modification Analysis

DNA methylation data is from TCGA-PAAD Illumina 450K methylation microarray. The histone modification ChIP-seq data (H3K4me3, H3K27ac) were derived from GEO and ENCODE [20] (data accession: GSM3376452, GSM2466034, GSM1574235, ENCSR520BIM, GSM2700597, ENCSR-596PFU, GSM945261, GSM2286771, GSM1574256, ENCSR876DCP, and ENCSR554RQQ). The Cistrome Data Browser [21], which has one pipeline to process all containing ChIP-seq data, was used to link WashU Browser online visualization.

## 3. Results

### 3.1. Preparation of Clinical Pancreatic Disease Datasets and Construction of the Workflow

A total of 875 datasets covering 555 patients with available survival data were included in this study (Table 1). The dataset of GSE28735 was used for the selection of differentially expressed genes (DEGs) and functional enrichment analysis. For this, we used the TCGA-PAAD dataset, which contained 177 PC patients with detailed survival data, combined with the selected DEGs to construct a gene expression signature prognostic risk score model based on the LASSO Cox regression. The GSE21501 (102 patients), Bailey et al., 2016 (96 patients), GSE57495 (63 patients), GSE62165 (131 patients), GSE62452 (66 patients), and GSE79668 (51 patients) cohorts were used for further validation. The workflow of the experimental strategy is shown in Supplementary Figure 1.

### 3.2. Selection of DEGs between Pancreatic Cancer and Adjacent Normal Tissues

**Figure 1:**
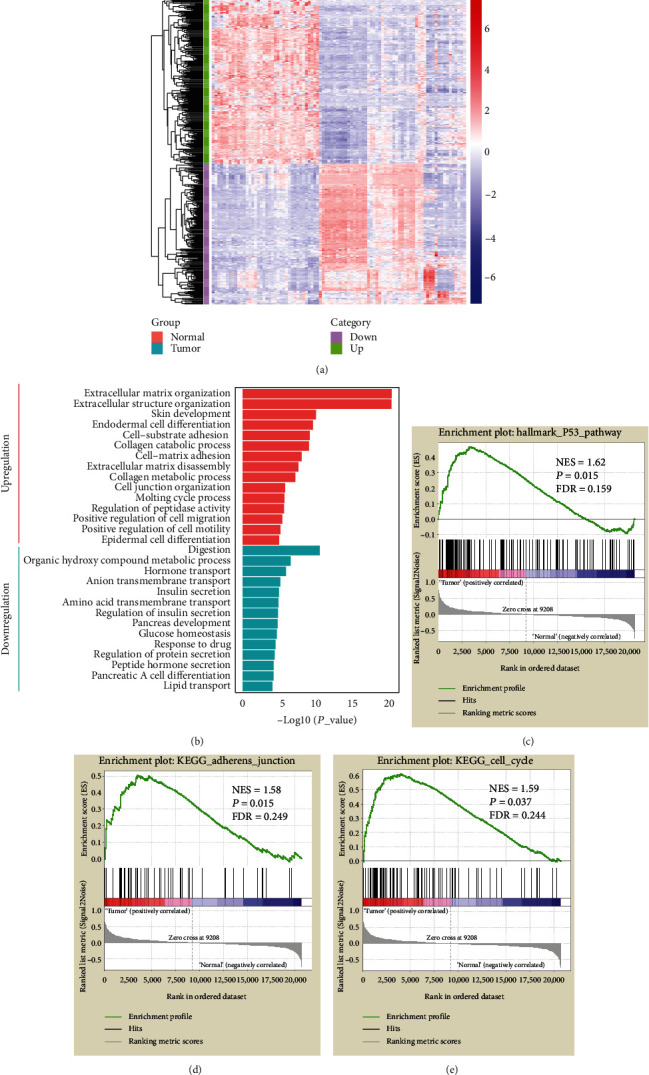
Identification of the genes differentially expressed between tumor and normal tissues. (a) Hierarchical clustering of DEGs in 45 paired PC (in green) and adjacent normal tissue samples (in red). Each row represents an individual differentially expressed gene, and each column represents an individual sample. Pseudocolors indicate relative expression levels from low to high on a log2 scale from -8 to 8; (b) GO biological process analysis for the upregulated and downregulated DEGs in Metascape online tool; (c–e) GSEA of the expression profile of the tumor samples in comparison to that of normal tissues from the GSE28735 dataset.

In order to select specific genes associated with pancreatic tumorigenesis, we first used the GSE28735 dataset, which contained the gene expression information of 45 pairs of pancreatic cancer and adjacent normal tissues. The supervised analysis compared the expression profiles of the 45 pairs of pancreatic cancer and adjacent normal tissues using the paired *t*-test in R. A false discovery rate (FDR) was applied to correct for the multiple testing hypothesis, and the significant genes were selected by the following threshold: FDR < 0.05, ∣log2 (fold change) | >1. The results indicated that 441 genes were differentially expressed between the two groups (Figure 1(a)). Of these, 238 upregulated genes were involved in cell adhesion and cell cycle by Gene Ontology (GO) analysis, while 203 genes related to secretion function were downregulated compared to the tumor with normal tissues. Further analysis by gene set enrichment analysis (GSEA) showed that the p53, cell cycle, cell adhesion junction, PI3K-AKT-mTORC, Notch, TGF*β*, epithelial-mesenchymal transition (EMT), and other cancer-related signaling pathways were enriched in the tumors, which was in accordance with previous studies (Figures 1(c)–1(e) and Supplementary Figure 2A-L).

### 3.3. Establishment of a Five-Gene Expression Signature in Pancreatic Cancer

**Figure 2:**
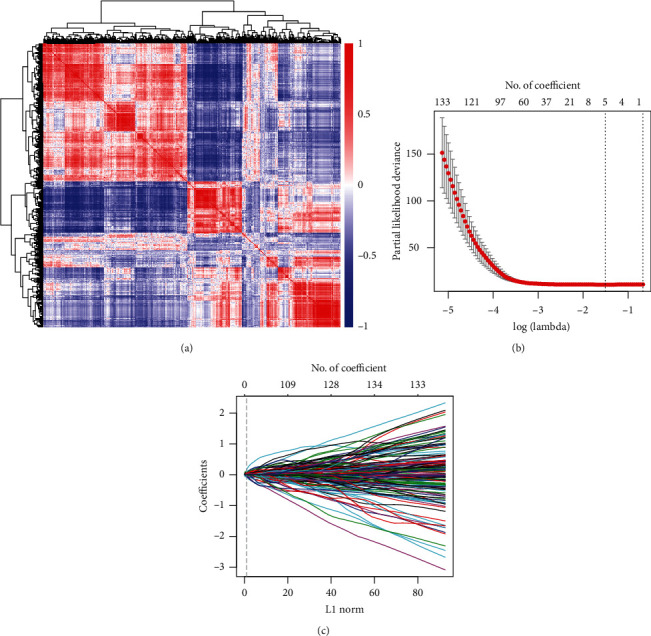
Establishment of a gene expression signature for overall survival prediction in the training cohort. (a) Hierarchical clustering shows the collinear expression of the DEGs. A correlation matrix heatmap of DEGs in the training cohort, in which each cell represents the Pearson correlation between the row and column of DEGs, the heatmap bar color along with the change of correlation coefficient from -1 to 1; (b, c) selection of the OS-associated genes by the LASSO Cox regression model. LASSO coefficient profiles of 441 differentially expressed associated genes. Each curve corresponds to a gene; the vertical line is drawn at the value lambda = 0.2215 chosen by 10-fold cross-validation.

**Figure 3:**
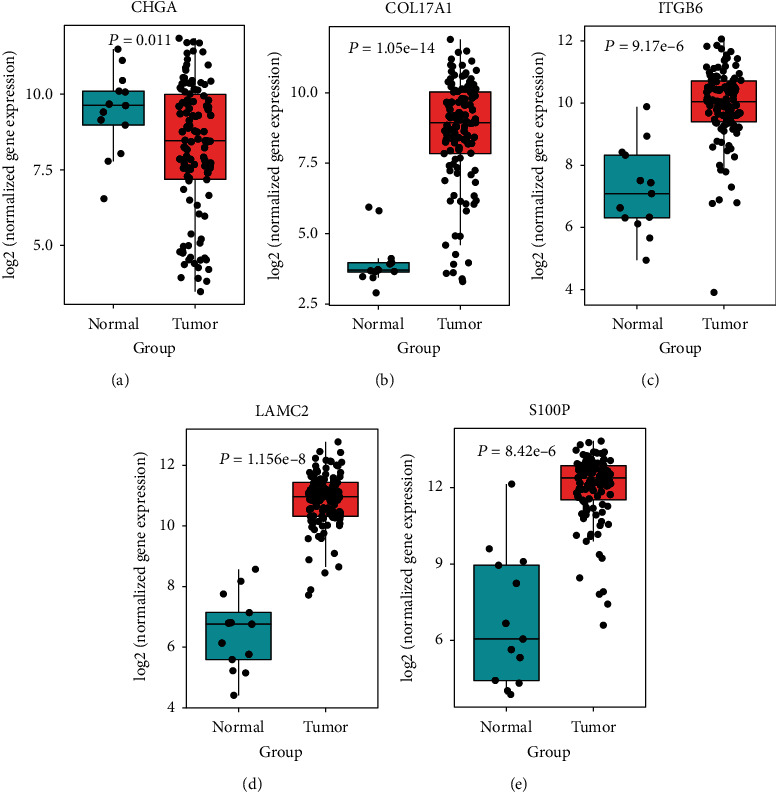
The expression of five genes in PC and normal tissues. (a) *CHGA*; (b) *COL17A1*; (c) *ITGB6*; (d) *LAMC2*; (e) *S100P*. *P* values were calculated by a *t*-test on the log2 RMA normalization of the expression data.

**Figure 4:**
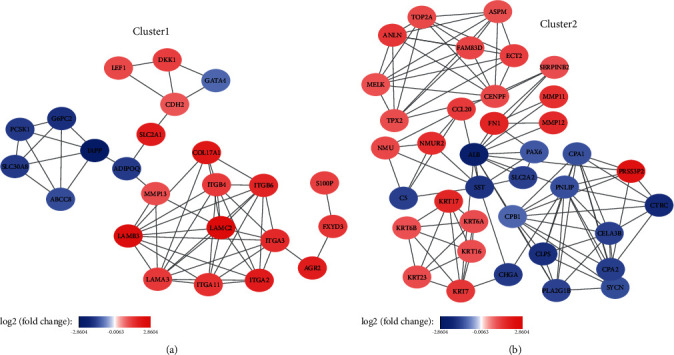
Five genes of protein-protein interaction (PPI) networks clustered by MCODE algorithm. (a) Cluster 1 module contains COL17A1, ITGB6, LAMC2, and S100P protein; (b) Cluster 2 module contains CHGA protein. The color of a node in the PPI network reflects the log2 (fold change) value of the gene expression.

To identify the mRNAs associated with OS in PC patients, we downloaded the transcriptome data from TCGA, which contained 177 patients with detailed survival information, for further investigation. The DEGs were merged with TCGA transcriptome data to form the training dataset. We observed collinearity among the DEGs (Figure 2(a)) in the training cohort, which would prejudice the results of traditional Cox regression analysis. Therefore, the LASSO Cox regression model selects the prognostic mRNAs to predict the survival-associated genes (Figures 2(b) and 2(c)). Finally, five genes out of 441 DEGs were selected: *CHGA*, *COL17A1*, *ITGB6*, *LAMC2*, and *S100P* (Table 2). Among these, *COL17A1*, *ITGB6*, *LAMC2*, and *S100P* were upregulated in tumor tissues, whereas only *CHGA* was downregulated. The five-gene expression levels were further validated in an independent cohort (GSE62165), which contains 118 neoplastic and 13 normal tissues. There was a significant differential expression in pancreatic cancer patients compared to normal ones (*P* < 0.001), suggesting that they might be a potential biomarker signature for pancreatic patients (Figures 3(a)–3(e)). Next, in order to explore the interaction network of the identified gene signature, we generated a protein-protein interaction network (PPI) from DEGs using the STRING online tool. Clustered by MCODE algorithm [22] in Cytoscape [23], two modules with the five-gene signature were selected (Figure 4); each module protein might form a large complex to regulate some biological process. The GO and KEGG analysis showed that the Cluster 1 genes were significantly enriched in the cell adhesion and ECM-receptor interaction signal pathways, while the Cluster 2 genes participated in the pancreatic secretion process (Supplementary Figure 3).

### 3.4. Construction of Prognostic Risk Score Model for Long-Term Survival Prediction

**Figure 5:**
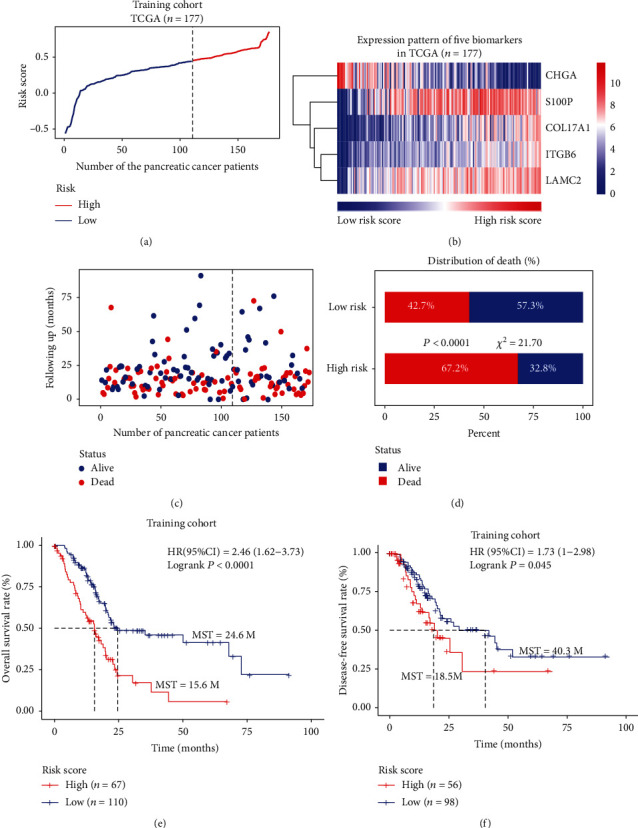
The five-gene signature-based risk score in the prognosis of survival in the training cohort. (a) Risk score distribution of the final five-gene expression signature in high- and low-risk groups; (b) the expression profiles of the five-gene signatures from low- to high-risk score; (c) the vital survival status of patients in the high- and low-risk cohorts; (d) distribution of mortality rate in high- and low-risk score groups. (e) Kaplan-Meier analysis with log-rank test for overall survival of the PC patients in the high- and low-risk score groups; (f) Kaplan-Meier analysis with log-rank test for disease-free survival of the PC patients in the high- and low-risk score groups.

Based on the expression levels of these five genes, the following risk score formula was generated for further evaluation from the TCGA training cohort: Risk score = −0.0481∗CHGA + 0.0402∗COL17A1 + 0.0697∗ITGB6 + 0.0021∗LAMC2 + 0.0063∗S100P. Using this formula, PC patients in the training cohort were divided into high- and low-risk score subgroups according to the optimal selected cutoff score (0.46) calculated by X-tile plots [16] based on their association with the OS (Supplementary Figure 4).  Figure 5(a) indicates the division of PC patients into high- and low-risk groups by this formula, and Figure 5(b) shows the expression patterns of the five genes from low- to high-risk score groups. The results indicated that the distribution of mortality in the high-risk group was significantly higher than that in the low-risk group (67.2% vs. 42.7%, *P* < 0.0001, Figures 5(c) and 5(d)). In addition, Kaplan-Meier analysis indicated that patients with high- and low-risk scores had a median OS time of 15.6 months and 24.6 months, respectively (HR = 2.46, 95%CI = 1.62‐3.73, *P* < 0.0001, Figure 5(e)), and the median disease-free survival time was 18.5 vs. 40.3 months (HR = 1.73, 95%CI = 1‐2.98, *P* = 0.045, Figure 5(f)). To know about the prognostic efficiency of our model with the survival time, we performed the time-dependent ROC curve analysis. The ROC 1-, 2-, and 3-year survival predicted by the risk score is depicted, with AUCs of 0.654 (1-year), 0.615 (2-year), and 0.651 (3-year), respectively (Supplementary Figure 5A). These results imply that the five-gene expression signature has relatively high sensitivity and specificity in predicting the OS of PC patients.

### 3.5. External Validation of the Five-Gene Prognostic Signature with Different PC Datasets

**Figure 6:**
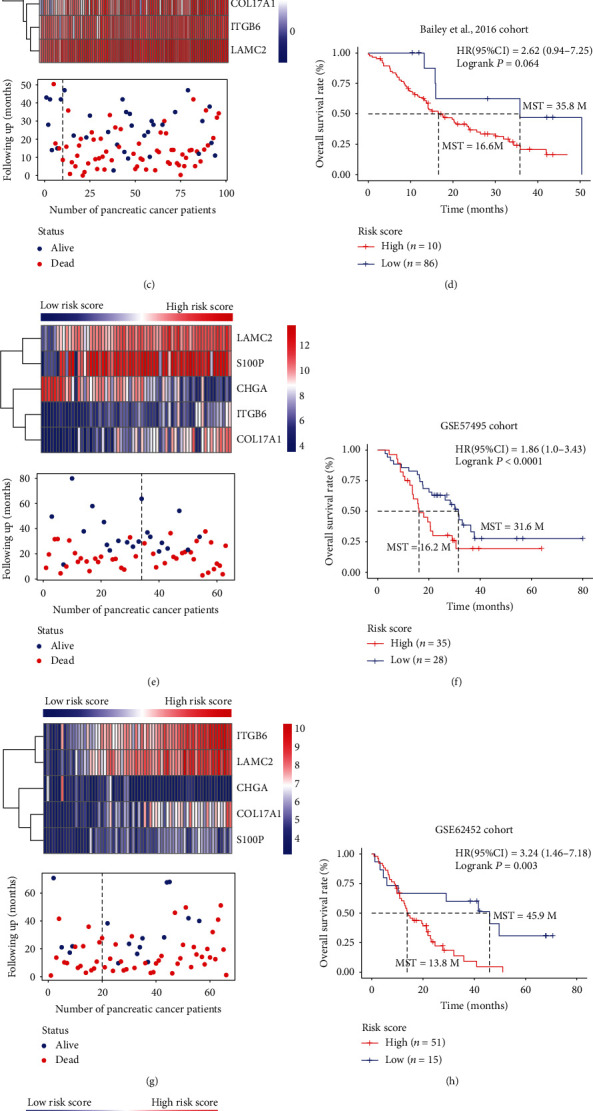
Performance assessment of the five-gene expression signature model in another five external independent validation datasets. (a, c, e, g, i) The heatmap and distribution of the five-gene expression profiles from low- to high-risk scores for the five external independent validation cohorts. (b, d, f, h, j) Kaplan-Meier overall survival analysis with log-rank test for the PC patients in high- and low-risk groups in five external independent validation cohorts.

To further validate the efficiency of this five-gene expression signature, we applied the formula and cutoff to five external independent validation datasets. Patients of each cohort were then divided into high- or low-risk subgroups. In the GSE21501 cohort, the five-gene signature expression pattern and OS analysis were similar to those in the training cohort (*P* < 0.001, HR = 2.61, 95%CI = 1.47‐4.62, Figures 6(a) and 6(b)). In the Bailey et al., 2016 cohort, the OS of patients discriminated by the gene expression signature was not statistically significant between the high- and low-risk groups (*P* = 0.064, HR = 2.62, 95%CI = 0.94‐7.25, Figures 6(c) and 6(d)). These hazard ratio values indicate that the gene expression signature could still be a potential risk factor in this cohort. Three other GEO datasets were also independently validated; the overall median survival time of high- and low-risk groups in the GSE57495 cohort was 16.2 months and 31.6 months, respectively (HR = 1.86, 95%CI = 1.0‐3.43, *P* = 0.047, Figures 6(e) and 6(f)). In the GSE62452 cohort, he median survival time of high- and low-risk groups was 13.8 months and 45.9 months (HR = 3.24, 95%CI = 1.46‐7.18, *P* = 0.003, Figures 6(g) and 6(h)), and in the GSE79668 cohort, they were 16.5 months and 96.9 months, respectively (HR = 4.44, 95%CI = 1.83‐10.73, *P* = 0.0009, Figures 6(i) and 6(j)). Time-dependent ROC was conducted in validation dataset analysis, showing a robust model constructed from our five-gene signature (Supplementary Figure 5B-E). The above results indicate the high predictive efficiency of this five-gene expression signature in PC patients.

### 3.6. Univariate and Multivariate Analysis Combined with Stratified Survival Analysis

**Figure 7:**
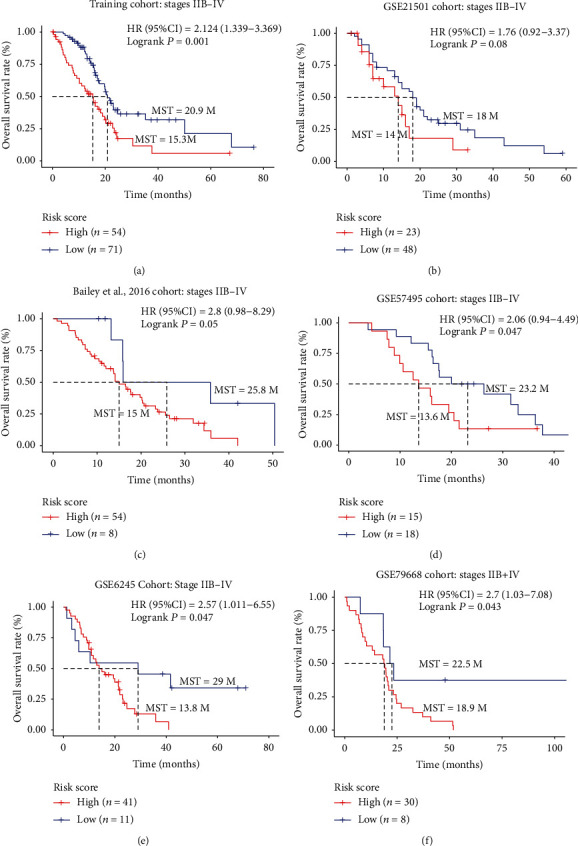
The association between five-gene signature and overall survival in patients with stages IIB-IV. The Kaplan-Meier survival curve of the training cohort (a) and the five external independent validation cohorts (b–f).

**Figure 8:**
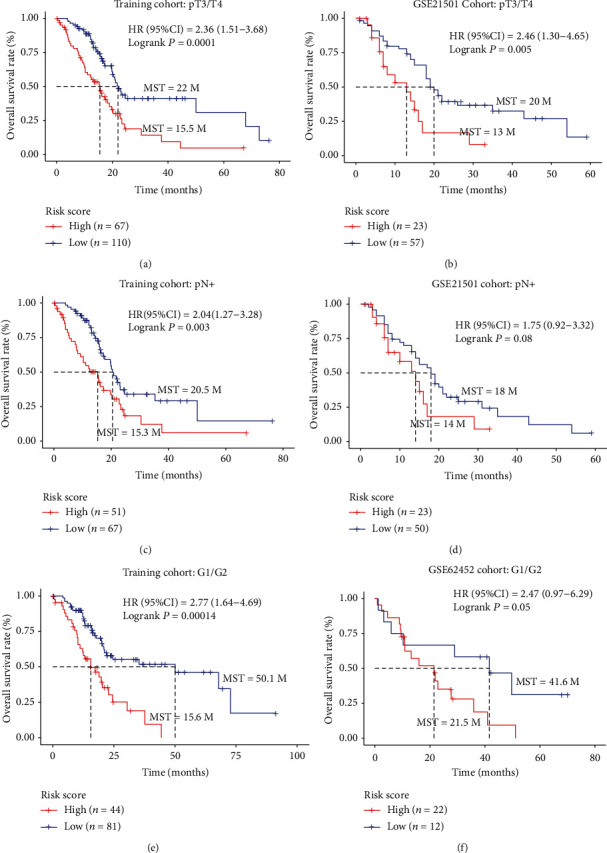
The five-gene signature was associated with prognosis in patients with advanced-stage cancer. (a, b) Kaplan-Meier analysis of the OS of patients with T3/4 stage in the training cohort and GSE21501 cohort; (c, d) Kaplan-Meier analysis of the OS of patients with lymph node metastasis in the training cohort and GSE21501 cohort; (e, f) Kaplan-Meier analysis of the OS of patients with grade 1/2 stage in the training cohort and GSE62452 cohort.

Univariate and multivariate survival analysis was performed on the five-gene signature and clinicopathological features for OS. We found that the five-gene signature was an independent prognostic factor of PC patients between the training and external independent cohorts (Table 3, Supplementary Table 1). The univariate analysis showed that the AJCC, T, and N stages and histological grade had relatively significant impacts on prognosis. Therefore, we performed stratified survival analysis by the individual clinicopathological features to evaluate the prognostic values of our risk score model in the training cohort and external independent datasets. According to the results of stratified analysis (Supplementary Table 2), we concluded that this signature pattern could be further used to discriminate those patients in the relatively late-stage AJCC IIB-IV stages (Figure 7), T3/T4 tumors (Figures 8(a) and 8(b)), lymph node metastasis (Figures 8(c) and 8(d)), and lower-grade tumor G1 and G2 tumors (Figures 8(e) and 8(f)). This observation indicates that the five-gene expression signature also could be applied in clinicopathological subgroups, which, to some extent, indicated the reliability and general applicability of our risk score model.

### 3.7. Epigenetic Regulation of the “Five Genes” in PC

**Figure 9:**
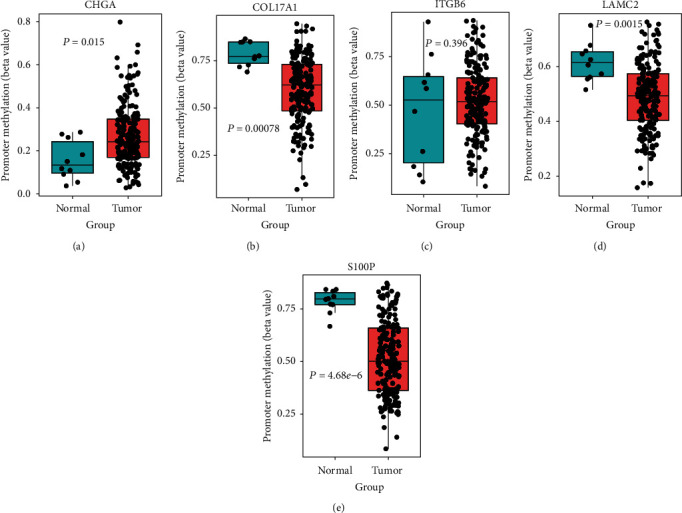
Promoter methylation status of five genes between PC and normal tissues in TCGA Illumina 450K methylation dataset: (a) *CHGA*; (b) *COL17A1*; (c) *ITGB6*; (d) *LAMC2*; (e) *S100P.*

**Figure 10:**
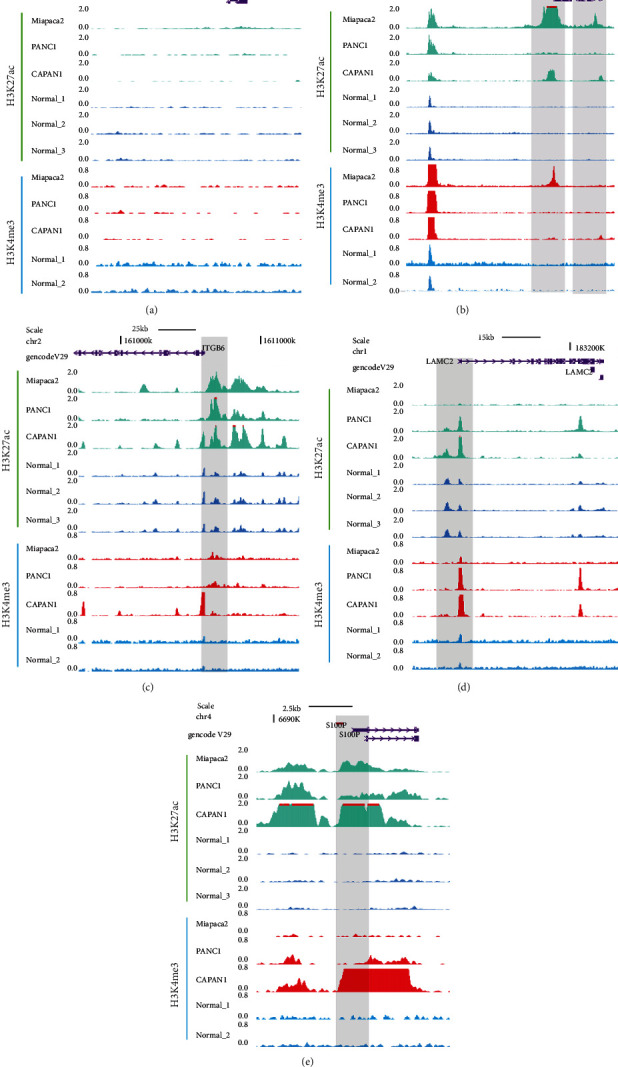
WashU Epigenome browser view of activated (H3K27ac and H3K4me3) histone modification tracks showing the promoter region around *CHGA* (a), *COL17A1* (b), *ITGB6* (c), *LAMC2* (d), and *S100P* (e) in pancreatic cancer cell line (Miapaca2, PANC1, and CAPAN1) and normal tissue cells.

In order to clarify the mechanism underlying the expression pattern of these five genes, and given that epigenetic modifications are highly related to tumorigenesis, we examined their epigenetic regulation by comparing promoter DNA methylation and histone modification markers of the five genes in pancreatic tumor and healthy cells. DNA methylation analysis showed that *COL17A1*, *LAMC2*, and *S100P* gene promoter methylation was downregulated (Figures 9(b), 9(d), and 9(e)), while *CHGA* gene promoter methylation was significantly upregulated (Figure 9(a)), which was in accordance with the gene expression pattern (Figure 3). Although methylation of the *ITGB6* promoter was not obviously different (Figure 9(c)), the activated histone markers, H3K27ac and H3K4me3, were significantly upregulated in the *ITGB6* promoter in the tumor cells (Figure 10(c)). Likewise, the promoter regions of the other upregulated genes, *S100P*, *COL17A1*, and *LAMC2*, were also associated with the activated chromatin state (Figures 10(b), 10(d), and 10(e)), while the downregulated gene *CHGA* lacked activated histone modification (Figure 10(a)). These observations presumably elucidate that the differential expression of these genes between healthy and PC cells was coregulated by multiple epigenetic factors.

## 4. Discussion

Clinical histopathological parameters, such as TNM stage and the level of tumor differentiation, are currently used for prognostic prediction of PC patients. However, this system has obvious limitations due to the lack of understanding of tumor heterogeneity. Genetic molecular subtyping of PC is only in its infancy, but current progress has already shown its potential value to discriminate patients into different subtypes, related to very different OS and therapeutic response. Along the same line, based on the development of cancer genomics, use of gene expression signatures for clinical prediction has also made great progress. Haider et al. established a 36-gene expression signature for prognosis with satisfactory results [24]. Klett et al. reported a 17-gene subset that could be applied for prognostic evaluation and early diagnosis and could discriminate pancreatic cancer from nontumor tissues, pancreatic precursor lesions, and pancreatitis [25]. Currently, blood-based CA 19-9 is widely used for diagnosis and prognosis in PC patients; however, due to its low sensitivity and specificity, clinical prediction is not satisfactory. Furthermore, there are CA 19-9-negative patients due to limited Lewis antigen [26].

Genetic sequencing offers a new approach to precision medicine. Moreover, current targeted cancer therapy has essentially been established on the results of studies about gene detection [27–29]. We therefore attempted to construct a gene expression signature for prognostic assessment. This study was built on a clinical problem: some early-stage PC patients do not show a favorable survival rate even in comparison to the OS of late-stage PC patients who underwent resection. This observation indicates that histopathological classification is not sufficient for the prognostic and therapeutic assessment. We hypothesized that molecular differences between the samples categorized into the same groups by traditional approaches might be the underlying reason. Thus, prognostic assessment using a gene expression signature could allow patients to avoid unnecessary or even detrimental treatment modalities, such as operations, or chemotherapy. The ideal prognostic model should have high prediction efficiency with as few genes as possible, to increase clinical practicality. Therefore, we first selected PC-associated genes by screening the differentially expressed genes between 45 pairs of PC and adjacent healthy tissues. Next, 177 PC patients with recorded survival information were used as the training cohort to construct the prognostic model by LASSO Cox regression. Finally, a five-gene expression signature with a risk score equation was constructed. In order to test the efficiency of this signature, several other datasets were used as the validation cohorts. The results were also highly positive given that this signature correlated with DFS and discriminated patients in several different cohorts into high- and low-risk groups, who also had different prognoses. These results indicated that the prognosis of some patients in the high-risk groups was poor, and these patients even belonged to an early-stage category, such as IIA. In lymph node-positive, T3, and T4 PC patients, the low-risk groups still had a relatively better prognosis compared to that of the high-risk groups. These results were congruent with our hypothesis that traditional histopathological and blood-based CA 19-9 approaches were insufficient for prognostic evaluation compared with the genetic classifiers when tumor heterogeneity is taken into consideration.

Although this five-gene expression signature was tested successful in different cohorts which contained hundreds of PC patients, the potential mechanism affecting the expression of these genes was still unclear. CHGA is a member of the chromogranin/secretogranin family of neuroendocrine secretory proteins found in the secretory vesicles of neurons and endocrine cells. It is involved in pancreatic beta cell secretion, negative regulation of insulin, and hormone secretion (Figure 1(b)). In recent years, some previous works have revealed *CHGA* as a novel biomarker for PC [30–32]. *ITGB6* and *LAMC2* had been reported to be associated with activation of the EMT, cell adhesion, TGF*β*, PI3K-AKT, and MAPK pathways [33–39], which are all involved in PC tumorigenesis. Additionally, these pathways were also enriched in our study (Figure 1 and Supplementary Figure 2). COL17A1 is a transmembrane protein, which mediates cell adhesion and extracellular matrix organization. It is underexpressed in breast cancer and overexpressed in cervical and other epithelial cancers. The *COL17A1* promoter methylation status accurately predicts both the direction of misexpression and the increasingly invasive nature of epithelial cancers [40]. Our work also implied that *COL17A1* was overexpressed and its promoter displayed aberrant DNA methylation in PC compared to that in adjacent healthy tissues (Figures 3(b) and 9(b)). *S100P* had been revealed to be related to increased cancer cell invasion and metastasis in PC [41, 42], and Matsunaga et al. had found S100P presence in the duodenal fluid to be a useful diagnostic marker for pancreatic ductal adenocarcinoma [43]. Next, we looked into the involvement of epigenetic modifications. Without altering the genetic sequence, epigenetic modification can regulate gene expression at the transcriptional and posttranscriptional levels, which has become the main target for cancer therapy [44]. We found that H3K27ac and H3K4me3 modification was significantly different between pancreatic cell lines and normal pancreatic tissues (Figures 10(b)–10(e)), and these epigenetic differences can cause differences in the expression levels of these genes in tumor and adjacent healthy tissues. Furthermore, epigenetic inhibition is currently a potential cancer therapy, and some inhibitors have already been approved for some types of cancer, such as vorinostat in T-cell lymphoma and bortezomib in melanoma [45, 46]. Although some of these five prognosis-associated genes had previously been used for therapeutic purposes, their epigenetic regulations were unknown. Our results could offer new insight for identifying new therapeutic targets.

There are still some limitations to our study. Firstly, clinical parameters such as gender, age, medical history, (neo)adjuvant chemotherapy, or radiotherapy were not always complete; thus, we could not evaluate the relationship between the gene expression signature with these parameters in all the datasets. Furthermore, the input for our study was derived from public databases, and hence, our study is retrospective. Validation with a prospective study is needed. We are currently in the process of evaluating this gene expression signature in blood, urine, and saliva samples in order to clarify whether this signature can be used for early detection through these routes. Simultaneously, we are working on the identification of a gene expression signature to be used in patients undergoing chemotherapy.

## 5. Conclusion

Taken together, we established a novel model for robust biomarker identification for PC. Subsequent analysis and review of previous works revealed the diagnostic and prognostic influence of the five-gene signature on PC. In the future, we believe that therapeutic targeting of specific genes will be an effective method.

## Figures and Tables

**Table 1:** Characterization of the included datasets.

Datasets	Study	Platform	Cases	Description
GSE28735	Zhang et al. 2012	Affymetrix HG-1.0 ST	90	45 pairs of tumor with adjacent healthy tissues
GSE21501	Stratford et al. 2010	Agilent-014859 WHG 4X44K	132	102 patients with survival data
GSE57495	Chen et al. 2015	Affymetrix	63	63 patients with survival data
GSE79668	Kirby et al. 2016	RNAseq, Illumina Hiseq2000	51	51 patients with survival data
*Bailey*_ICGC_PACA_AU	Bailey et al. 2016	RNAseq Illumina	96	96 patients with survival data
GSE62165	Janky et al. 2016	Affymetrix HG-U219	131	118 surgically resected PDAC and 13 healthy tissues
GSE62452	Yang et al. 2014	Affymetrix HG-1.0 ST	130	66 patients with survival data; 69 tumors + 61 nontumors
TCGA	TCGA_PAAD_UCSC_Xena	RNA Illumina v3	182	177 patients with survival and clinical data

**Table 2:** Characterization of the five candidate genes in hg 19 genome.

Gene symbol	Description	Expression status	Coordinate	*P* value^a^	*χ* ^2^ ^b^	Coefficient^c^
*CHGA*	Chromogranin A	Down tumor/normal	Chr14:93389445-93401638	<0.0001	18.35	-0.0481
*COL17A1*	Collagen type XVII alpha 1 chain	Up tumor/normal	Chr10:105791046-105845638	<0.0001	21.05	0.0402
*ITGB6*	Integrin subunit beta 6	Up tumor/normal	Chr2:160956182-161110349	<0.0001	16.13	0.0697
*LAMC2*	Laminin subunit gamma 2	Up tumor/normal	Chr1:183147952-183214262	0.00015	14.44	0.0021
*S100P*	S100 calcium binding protein P	Up tumor/normal	Chr4:6695566-6698897	0.0023	9.256	0.0063

^a^Derived from the univariate Cox proportional hazards regression analysis in the training cohort (log-rank test). ^b^Derived from the univariate Cox proportional hazards regression analysis in the training cohort (Chi^2^ test). ^c^Derived from the LASSO Cox regression analysis coefficients in the training cohort.

**Table 3:** Univariate and multivariate Cox regression analysis of five-gene signature and clinicopathological characteristics with overall survival in the training and another two external validation datasets.

Variable	Training (TCGA) cohort					GSE21501 cohort					GSE79668 cohort				
	*N*	Univariate		Multivariate		*N*	Univariate		Multivariate		*N*	Univariate		Multivariate	
		*P*	HR (95% CI)	*P*	HR (95% CI)		*P*	HR (95% CI)	*P*	HR (95% CI)		*P*	HR (95% CI)	*P*	HR (95% CI)
*AJCC stage*															
I-IIA vs. IIB-IV	48/125	0.012	1.93 (1.15-3.24)	0.68	1.37 (0.30-6.27)	26/71	0.044	1.83 (1.0-3.29)	0.059	1.83 (0.97-3.42)	13/38	0.25	1.52 (0.74-3.1)	0.98	0.97 (0.11-8.86)
*T stage*															
T1/T2 vs. T3/T4	31/144	0.03	2.02 (1.07-3.81)	0.87	1.06 (0.51-2.23)	18/80	0.85	0.94 (0.51-1.74)	0.24	0.68 (0.36-1.30)	15/36	0.05	1.97 (0.97-3.98)	0.36	1.48 (0.63-3.49)
*N stage*															
N0 vs. N+	50/118	0.003	2.16 (1.28-3.65)	0.58	1.48 (0.36-6.00)	28/73	0.035	1.83 (1.04-3.22)	0.087	1.74 (0.92-3.28)	14/37	0.29	1.44 (0.72-2.87)	0.96	0.97 (0.12-7.69)
*Grade*															
G1/G2 vs. G3/G4	125/50	0.05	1.54 (0.99-2.37)	0.53	1.17 (0.72-1.89)										
*Gender*															
Female vs. male	80/97	0.31	1.24 (0.82-1.86)	0.51	0.86 (0.55-1.35)						19/32	0.501	1.24 (0.66-2.30)	0.87	1.05 (0.55-2.01)
*Age (years)*															
>60 vs. ≤60	58/119	0.12	1.42 (0.90-2.24)	0.13	1.49 (0.89-2.51)						18/33	0.35	1.34 (0.72-2.49)	0.61	1.19 (0.61-2.31)
*Signature*															
High vs. low risk	67/110	<0.0001	2.46 (1.62-3.73)	0.002	2.05 (1.29-3.24)	27/75	<0.001	2.61 (1.47-4.62)	0.005	2.31 (1.28-4.18)	38/13	0.0009	4.44 (1.83-10.73)	0.0044	3.84 (1.52-72)

## Data Availability

The datasets supporting this study can be found in the GEO (https://www.ncbi.nlm.nih.gov/gds/) and the UCSC-Xena browser (http://xenabrowser.net/datapages/) repository.

## Supplementary Materials

Supplementary Materials Supplementary Table 1: univariate and multivariate Cox analysis in external three independent datasets. Supplementary Table 2: stratified survival analysis of clinical clinicopathological characteristics of training and validation cohorts. Supplementary Table 3: statistics information of the datasets included in this study. Supplementary Figure 1: the workflow of construction and evaluation of our prognostic model. Supplementary Figure 2: GSEA of the hallmarks and KEGG pathway between PC and adjacent normal tissues. A-G: the hallmarks enrichment of PC; H-L: KEGG pathway enrichment in PDAC. Supplementary Figure 3: GO term and KEGG pathway analysis for five genes related to protein network annotation. A: Cluster 1 contains COL17A1, ITGB6, LAMC2, and S100P; B: Cluster 2 contains CHGA. The barplot is derived from the Metascape online tool. Supplementary Figure 4: X-tile plots of the five selected gene expression signatures associated with the overall survival of the patients in the training cohort and with the LASSO risk values. Supplementary Figure 5: evaluation of gene expression signature-based risk score and robustness.
