# Supplementary material for: Establishment and Investigation of a Multiple Gene Expression Signature to Predict Long-Term Survival in Pancreatic Cancer
Source: Biomed Res Int. 2020 Sep 15;2020:1570862. doi: 10.1155/2020/1570862 (PMC7516738; doi:10.1155/2020/1570862)
Supplement: Supplementary Materials — Supplementary Table 1: univariate and multivariate Cox analysis in external three independent datasets. Supplementary Table 2: stratified survival analysis of clinical clinicopathological characteristics of training and validation cohorts. Supplementary Table 3: statistics information of the datasets included in this study. Supplementary Figure 1: the workflow of construction and evaluation of our prognostic model. Supplementary Figure 2: GSEA of the hallmarks and KEGG pathway between PC and adjacent normal tissues. A-G: the hallmarks enrichment of PC; H-L: KEGG pathway enrichment in PDAC. Supplementary Figure 3: GO term and KEGG pathway analysis for five genes related to protein network annotation. A: Cluster 1 contains COL17A1, ITGB6, LAMC2, and S100P; B: Cluster 2 contains CHGA. The barplot is derived from the Metascape online tool. Supplementary Figure 4: X-tile plots of the five selected gene expression signatures associated with the overall survival of the patients in the training cohort and with the LASSO risk values. Supplementary Figure 5: evaluation of gene expression signature-based risk score and robustness. [file 1570862.f1.pdf]

Supplementary Table 1. Univariate and multivariate Cox analysis in external three independent datasets.

**Variable**	**GSE57495 Cohort**					**Bailey2016 Cohort**					**GSE62452 Cohort**				
		Univariate		Multivariate			Univariate		Multivariate			Univariate		Multivariate	
	N	P	HR(95% CI)	P	HR (95%CI)	N	P	HR(95 %CI)	P	HR(95% CI)	N	P	HR(95% CI)	P	HR(95% CI)
**AJCC stage**															
**I-IIA vs IIB-IV**	30/33	**0.009**	2.34(1.23-4.43)	**0.005**	2.47 (1.29-4.70)	34/62	**0.014**	2.04 (1.15-3.62)	**0.0057**	2.42(1.29-4.54)	14 /52	0.13	1.73 (0.85-3.53)	0.08	1.94 (0.92-4.12)
**T stage**															
**T1/T2 vs T3/T4**															
**N stage**															
**N0 vs N+**															
**Grade**															
**G1/G2 vs G3/G4**						57/36	**0.015**	1.88(1.13- 3.14)	**0.043**	1.74(1.02-2.99)	34/31	**0.02**	2.02(1.12-3.67)	0.07	1.76(0.94-3.29)
**Gender**															
**Female vs Male**						47/49	0.75	1.08(0.66-1.78)	0.75	1.08(0.64-1.83)					
**Age(years)**															
**>60 vs <=60**						67/29	0.45	0.81(0.47-1.38)	0.64	1.14(0.64-2.04)					
**Signature**															
**High vs Low risk**	28/35	**0.047**	1.86(1.0-3.43)	**0.028**	1.99(1.07-3.69)	86/10	**0.064**	2.61(0.94-7.25)	**0.04**	2.84(1.0-8.05)	51 /15	**0.003**	3.24 (1.46-7.18)	**0.046**	2.38 (1.01-5.59)

Table S2. Stratified Survival analysis of clinical clinicopathological characteristics of training and validation cohorts

		Logrank P_value	High/Low	MST(High/Low)	MST(95%CI)	HR(95%CI)
**Training Cohort (TCGA)**	OS	**<0.0001**	67/110	15.6/24.6	11.1-19.9/20.5-NA	2.46 (1.62-3.73)
	DFS	**0.045**	56/98	18.5/40.3	12.1-NA/19.8-NA	1.73(1-2.98)
	Stage I-IIA	0.11	13/35	19.9/72.7	NA/9.97-NA	2.19(0.83-5.78)
	Stage IIB-IV	**0.001**	54/71	15.3/20.9	10.3-19.8/17.7-NA	2.12(1.34-3.37)
	pN+	**0.003**	51/67	15.3/20.5	9.23-20.2/17.23-35.3	2.04(1.27-3.28)
	pN-	0.08	14/36	19.9/72.7	13.1-NA/NA	2.35(0.9-6.13)
	pT1/2	0.7	5/26	19.9/NA	5.33-NA/17.73-NA	1.35(0.29-6.39)
	pT3/4	**0.0001**	62/82	15.5/22	10.3-19.8/19.9-NA	2.36(1.51-3.69)
	G1/G2	**0.00014**	44/81	15.6/50.1	11.1-24.4/21.1-NA	2.77(1.64-4.69)
	G3/G4	0.08	23/27	15.3/19.7	5.1-NA/15.7-NA	1.87(0.92-3.79)
	Age >60	**0.0071**	48/71	15.5/21.7	9.77-24.4/19.87-NA	1.97(1.20-3.21)
	Age <=60	**0.0006**	19/39	17.3/50.1	10.3-NA/50.1-NA	3.70(1.662-8.25)
	Male	**0.0001**	40/57	15.3/72.7	9.77-22.8/22.03-NA	3.17(1.73-5.80)
	Female	**0.045**	27/53	15.6/20.1	12.5-30.4/16.2-NA	1.83(1.01-3.32)
**GSE21501 Cohort**	OS	**0.001**	27/75	13/20	7-17/18/31	2.61(1.47-4.62)
	Stage I-IIA	**0.0022**	4/22	7/26	4-NA/22-NA	9.04(2.20-37.08)
	Stage IIB-IV	0.08	23/48	14/18	7-NA/14-25	1.76(0.92-3.37)
	pN+	0.08	23/50	14/18	7-NA/14-25	1.75(0.92-3.32)
	pN-	**0.001**	4/24	7/26	4-NA/20-NA	9.94(2.42-40.82)
	pT1/2	0.11	4/14	9.5/23	3-NA/8-NA	3.28(0.77-13.87)
	pT3/4	**0.005**	23/57	13/20	7-NA/18-43	2.46(1.30-4.65)
**Bailey2016 Cohort**	OS	0.064	86/10	16.6/35.8	13.8-23.8/15.9-NA	2.62(0.94-7.25)
	Stage I-IIA	0.99	2/32	0/30	NA/12.9	NA
	Stage IIB-IV	**0.05**	8/54	15/25.8	12-20.3/15.8-NA	2.8(0.98-8.29)
	G1/G2	0.143	50/7	20.7/50.4	14.3-42/35.8-NA	2.93(0.69-12.35)
	G3/G4	0.43	33/3	13.8/15.8	9.5-25.6/13.2-NA	1.77(0.42-7.53)
	Age >60	0.24	59/8	17.7/35.8	13.7-30/15.8-NA	1.84(0.65-5.19)
	Age <=60	0.9	27/2	14.1/NA	10.4-31.9/NA	NA
	Male	0.12	45/4	14.1/50.4	11.6-23.8/15.8-NA	5.20(0.71-38.2)

	Female	0.32	41/6	20.3/25.8	13.7-34.3/13.2-NA	1.86(0.55-6.28)
	OS	**0.047**	35/28	16.2/31.6	13.6-29.1/21.1-NA	1.86(1.0-3.43)
**GSE57495 Cohort**	Stage I-IIA	0.2	13/17	29.1/NA	15.6-NA/30.1/NA	1.95(0.69-5.47)
	Stage IIB-IV	0.07	15/18	13.6/23.2	9.93-21.5/16.43-NA	2.06(0.941-4.49)
	OS	**0.0037**	51/15	13.8/45.9	11.6-21.9/10.3-NA	3.24(1.46-7.18)
	Stage I-IIA	0.0873	10/4	13.9/49.7	9.5-NA/49.5-NA	4.08(0.81-20.52)
**GSE62452 Cohort**	Stage IIB-IV	**0.0474**	41/11	13.8/29	10.8-21.9/5.9-NA	2.57(1.01-6.55)
	G1/G2	**0.05**	22/12	21.5/41.6	10.9-NA/10.3-NA	2.47(0.97-6.29)
	G3/G4	0.32	29/2	13.7/25.2	9.8-21.3/4.5-NA	2.98(0.37-23.24)
	OS	**0.0009**	38/13	16.5/96.6	10.4-21/18.6-NA	4.44(1.84-10.73)
	Stage IIB-IV	**0.043**	30/8	18.9/22.5	18.6-NA/10.4-23.2	2.7(1.03-7.08)
**GSE79668 Cohort**	pN+	**0.044**	29/9	18.7/22.5	18.6-NA/10.4-23.3	2.68(1.02-7.05)
	pT3/4	0.122	29/7	18.1/21.6	10.4-21.4/18.4-NA	2.13(0.82-5.53)

Table S3. Statistics information of the datasets included in this study

	**Training (TCGA) Cohort**	**GSE21501 Cohort**	**Bailey2016 Cohort**	**GSE62452 Cohort**	**GSE79668 Cohort**	**GSE57495 Cohort**
Dataset Size	177	102	96	69	51	63
Age (%)						
<=60	119(67.2)	NA	29(43.3)	NA	19(37.3)	NA
>60	58(32.7)	NA	67(56.7)	NA	32(62.7)	NA
Gender (%)						
Male	97(54.8)	NA	49(51)	NA	32(62.7)	NA
Female	80(45.2)	NA	47(49)	NA	19(37.3)	NA
AJCC Stage (%)						
I/IIA	49(29.5)	26 (26.8)	34(35.4)	14(21.2)	13(25.5)	30(47.6)
IIB/III/IV	117(70.5)	71(73.2)	62(64.6)	52(78.8)	38(74.5)	33(52.7)
T stage (%)						
T1/T2	31(17.7)	18(18.4)	NA	NA	15(29.4)	NA
T3/T4	144(82.3)	80(81.6)	NA	NA	36(70.6)	NA
N stage(%)						
N0	50(30.1)	28(27.7)	NA	NA	14(27.5)	NA
N+	116(69.9)	73(72.3)	NA	NA	37(72.5)	NA
Grade(%)						
G1/G2	125(70.6)	NA	57(59.4)	34(52.3)	NA	NA
G3/G4	50(29.4)	NA	36(30.6)	31(47.7)	NA	NA
Death status (%)						
Yes	92(51.98)	67(65.7)	64(66.7)	50(75.8)	45(88.2)	42(66.7)
No	85(48.02)	35(34.3)	32(33.3)	16(24.2)	6(11.8)	21(33.3)

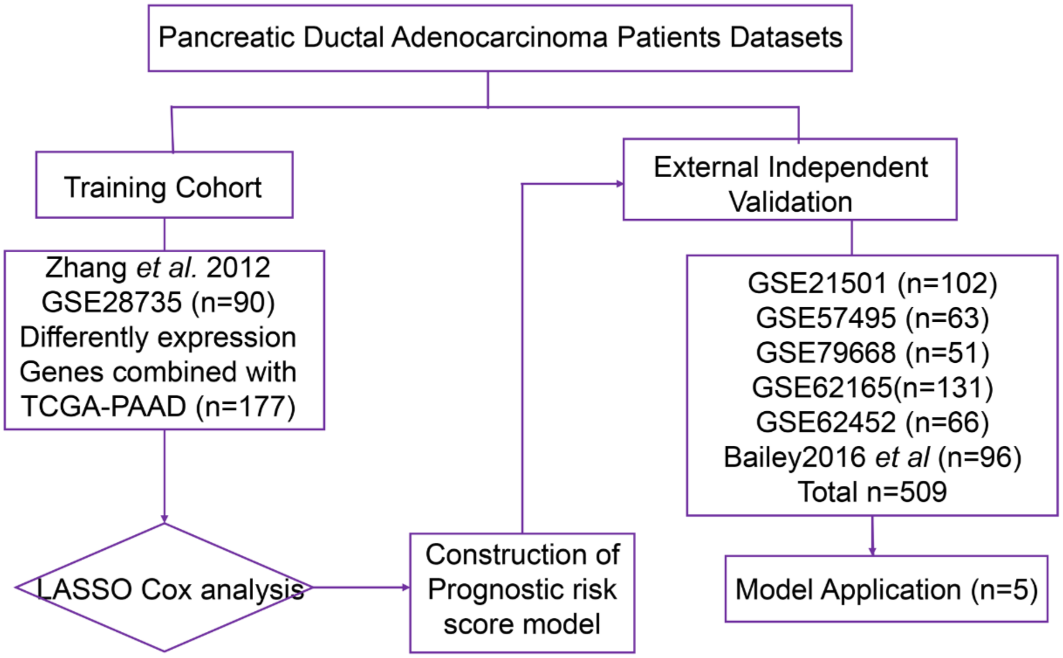

Supplementary Figure 1. The workflow of construction and evaluation of our prognostic model.

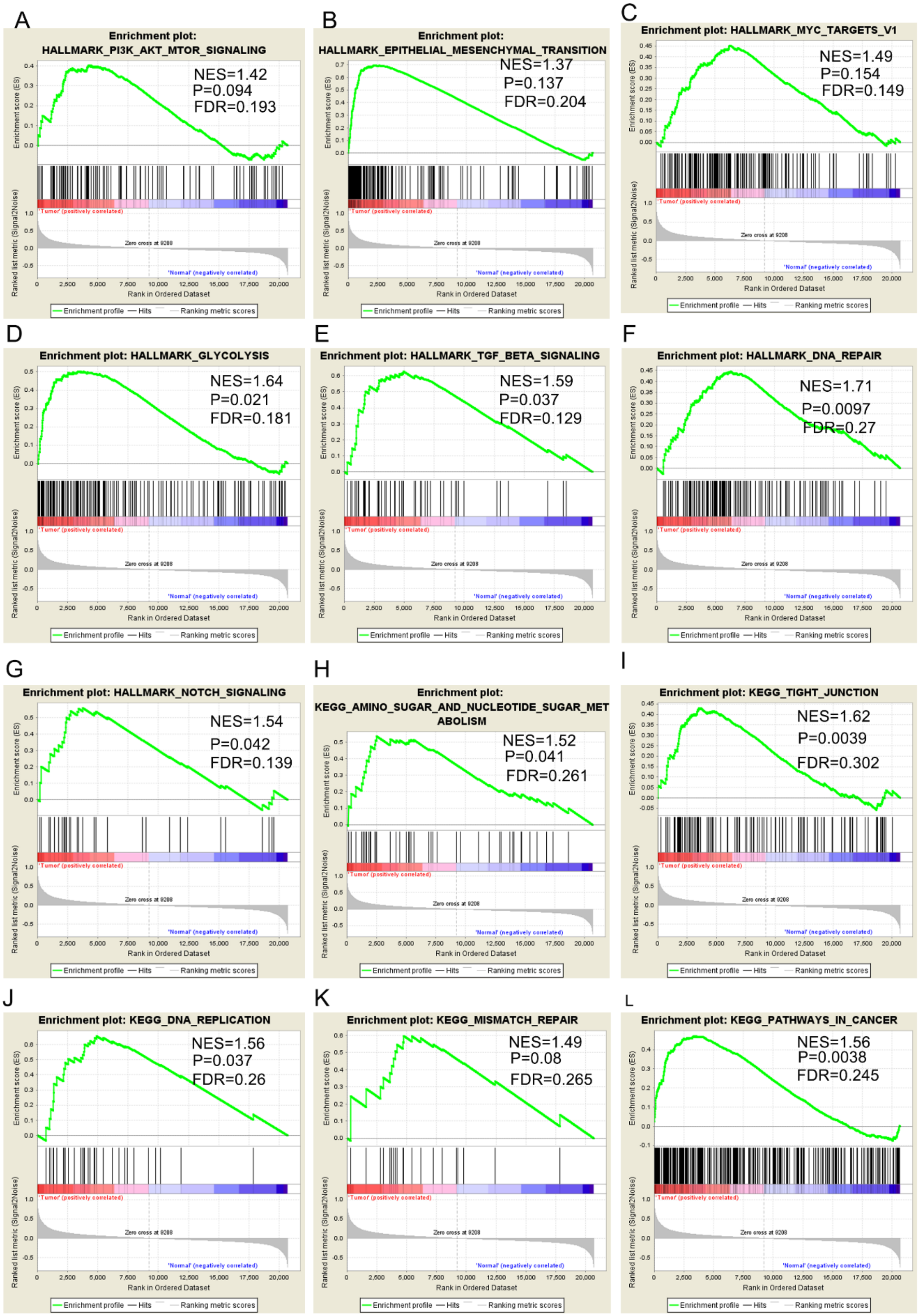

Supplementary Figure 2. GSEA analysis of the hallmarks and KEGG pathway between PC and adjacent normal tissues. A-G. the hallmarks enrichment of PC, H-L. KEGG pathway enrichment in PDAC.

A

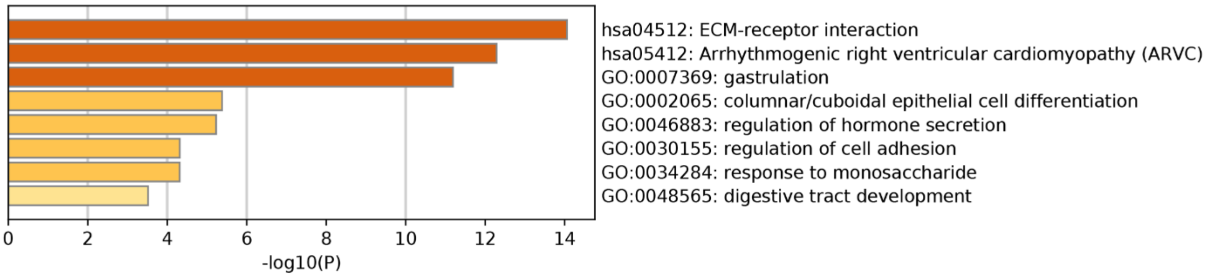

B

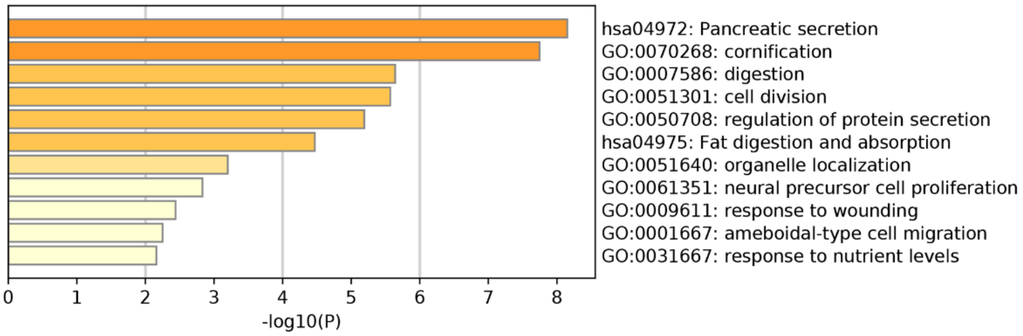

Supplementary Figure 3. GO Term and KEGG pathway analysis for five genes related to protein network annotation. A. Cluster 1 contains *COL17A1 ,ITGB6, LAMC2, S100P*; B. Cluster 2 contains with *CHGA*. The barplot is derived from Metascape online tool.

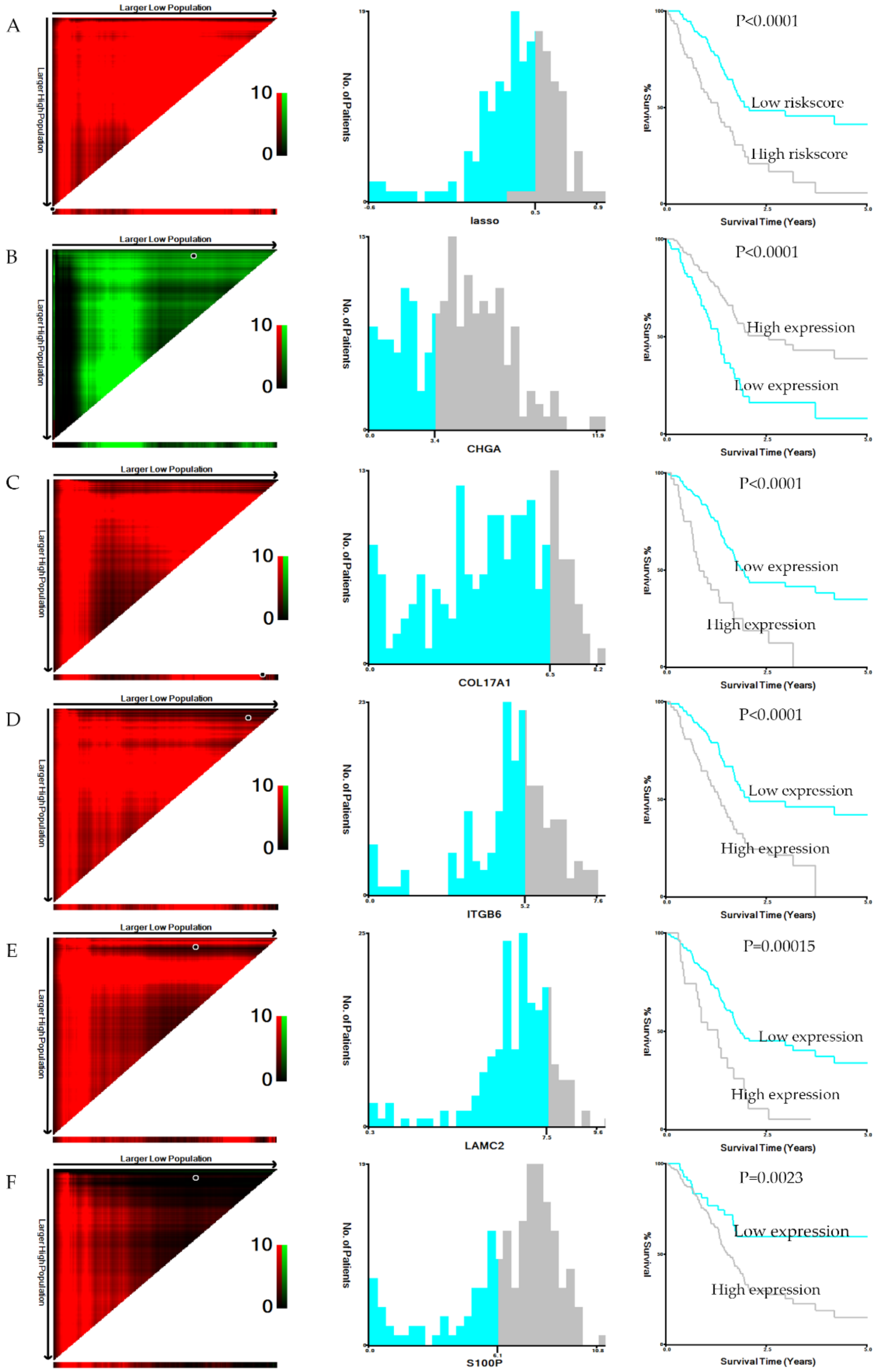

Supplementary Figure 4. X-tile plots of the five selected gene expression signatures associated with the overall survival of the patients in the training cohort, and with the LASSO risk values. Red represents inverse association between marker expression and survival, whereas green represents direct association (left panel). The value between green and gray in frequency histograms were the optimized cutoff value of high score and low score or high expression and low expression (median panel); overall survival between four genes and lasso risk score (right panel). High expression and

low expression of indicated gene expression signatures, high or low LASSO risk score derived from optimal cutoff value.

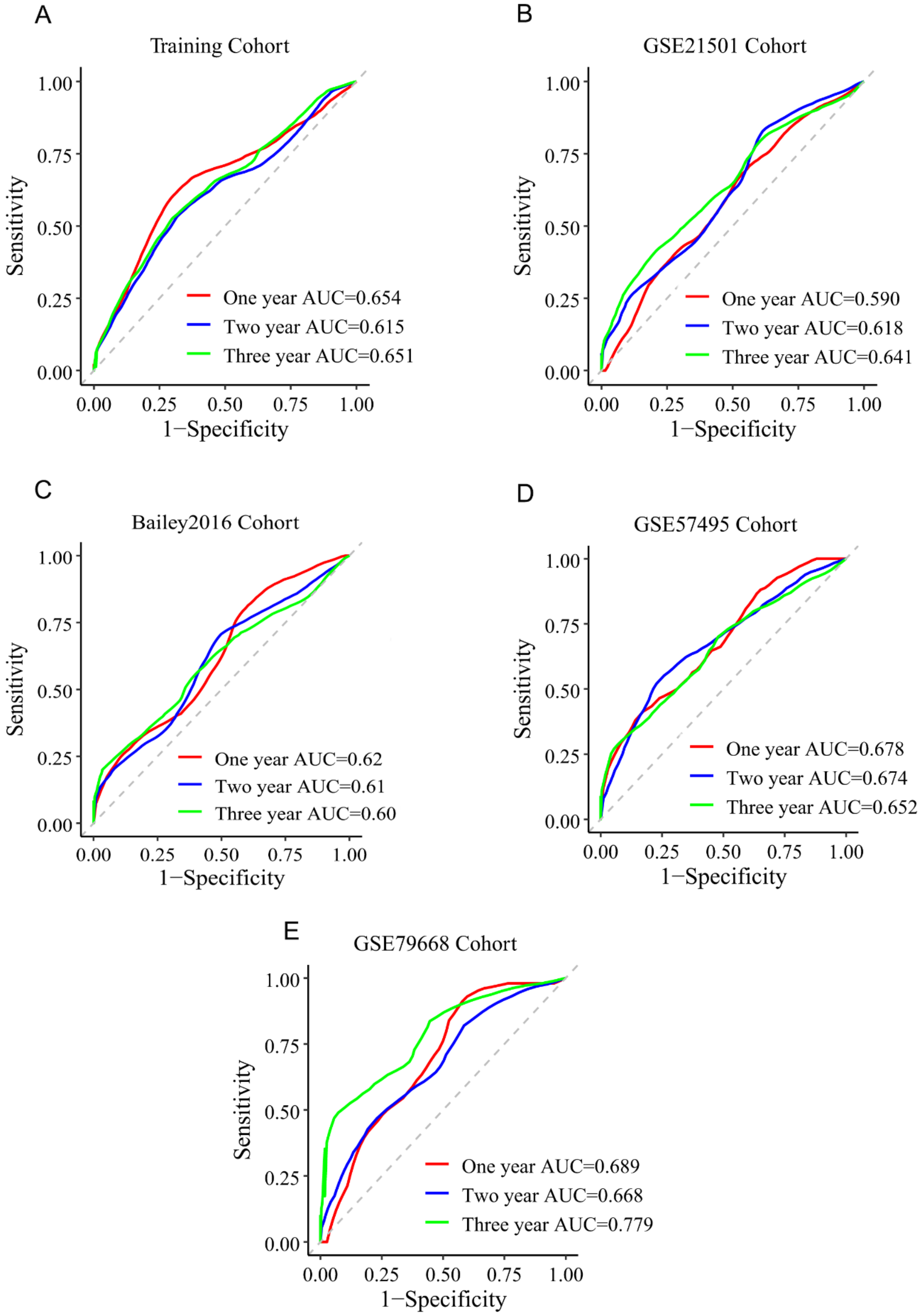

Supplementary Figure 5. Evaluation of gene expression signature-based risk score and robustness. Time-dependent ROC curves to assess the prognostic accuracy, AUC at 1, 2, and 3 years were

calculated according to the five-gene expression signature. A. Training cohort, B-E. external independent validation datasets.
